# Increased prevalence of nodular thyroid disease in patients with Klinefelter syndrome

**DOI:** 10.1007/s12020-023-03387-7

**Published:** 2023-05-06

**Authors:** Rosa di Fraia, Daniela Esposito, Lucia Digitale Selvaggio, Francesca Allosso, Roberto Alfano, Mario Rotondi, Giancarlo Balercia, Giacomo Accardo, Daniela Pasquali

**Affiliations:** 1 Department of Advanced Medical and Surgical Sciences, University of Campania, “L. Vanvitelli”, Naples, Italy; 2 Department of Endocrinology, Sahlgrenska Academy, University of Gothenburg, Gothenburg, Sweden; 3 Unit of Endocrinology and Metabolism, Laboratory for Endocrine Disruptors, RCCS Maugeri Clinical Scientific Institutes, Pavia, Italy; 4 Department of Internal Medicine and Therapeutics, University of Pavia, Pavia, Italy; 5 Department of Clinical and Molecular Sciences, University of Marche, Ancona, Italy; 6 Local Health Authority (ASL) Salerno, Salerno, Italy

**Keywords:** Thyroid, Thyroid nodules, Klinefelter syndrome, Hashimoto’s thyroiditis, 47, XXY

## Abstract

**Purpose:**

Thyroid dysfunction in patients with Klinefelter syndrome (KS) remains an unresolved issue. Although low free thyroxine (FT4) levels within the normal range and normal thyroid stimulating hormone (TSH) levels have been reported, there is currently no data on nodular thyroid disease in this population. This study aims to evaluate the results of thyroid ultrasound (US) examinations in KS patients compared with healthy controls.

**Methods:**

A cohort of 122 KS and 85 age-matched healthy male controls underwent thyroid US screening and thyroid hormone analysis. According to US risk-stratification systems, nodules ≥1 cm were examined by fine needle aspiration (FNA).

**Results:**

Thyroid US detected nodular thyroid disease in 31% of KS compared to 13% of controls. No statistical differences in the maximum diameter of the largest nodules and in moderate and highly suspicious nodules were found between patients and the control group. Six KS patients and two controls with nodules underwent FNA and were confirmed as cytologically benign. In line with published data, FT4 levels were found significantly near the lower limit of the normal range compared to controls, with no differences in TSH values between the two groups. Hashimoto’s thyroiditis was diagnosed in 9% of patients with KS.

**Conclusions:**

We observed a significantly higher prevalence of nodular thyroid disease in KS compared to the control group. The increase in nodular thyroid disease is likely linked to low levels of FT4, inappropriate TSH secretion, and/or genetic instability.

## Introduction

Klinefelter syndrome (KS) is the most frequently observed sex chromosome abnormality in men, with a frequency of 1:500 to 1:1000 [1–3]. Classical KS, accounting for 80–90% of cases, has a 47,XXY karyotype and presents with low serum testosterone, elevated gonadotropins, small and firm testes, azoospermia, and stature taller than the genetic target height [1, 2]. The clinical manifestations of KS are very often nuanced and heterogeneous, and this makes the diagnosis of KS challenging. Our understanding of KS, which is based on data collected since 1942, when the syndrome was first described, should be reassessed, and updated to better manage the condition [1, 3]. Several comorbidities are associated to KS, such as cardiovascular diseases, osteoporosis, metabolic syndrome, diabetes mellitus, and leg ulcers mostly related to hypogonadism [1–8]. In addition to testicular insufficiency KS is associated with several endocrine disorders, including thyroid dysfunction [1, 9–11]. However, no data are currently available regarding the frequency of thyroid nodules in KS. We conducted a PubMed search for English-language articles dealing with thyroid nodule management and KS from 1970 to January 2023, but no results were returned. A case report published in 1963, describes the incidental finding at postmortem of a nontoxic nodular goiter in a 71-year-old man with KS [12]. This report mentions that other authors had detected low iodine intake in three KS patients [13]. We previously performed a multicenter case-control evaluation based on data collected from the Klinefelter Italian Group (KING) database, which also assessed thyroid function in KS [2]. In this study, we evaluated the prevalence of thyroid diseases, the role of 47,XXY condition, and hypogonadism in thyroid dysregulation in KS patients and in non-KS hypogonadal men. FT4 was significantly lower in KS than in non-KS individuals, while TSH levels were similar. This finding was in line with previous studies suggesting subclinical hypothyroidism due to hypothalamic-pituitary dysfunction in KS [11, 14]. No evidence of an etiopathogenetic link to hypogonadal status or to a change in the set point of thyrotropic control was found. Whether patients with KS have increased risk of thyroid nodule disease needs to be filled, since patient management should be guided not only by the risk of malignancy, but also by the relative risks involved in any therapeutic intervention. Here, for the first time, we evaluated by high-resolution ultrasound (US) the frequency of undetected thyroid nodules in a cohort of adult KS patients compared to age-matched healthy men.

## Patients and methods

### Patient population

We performed a multicenter case-control study. The entire cohort included 155 consecutive KS patients and 95 age-matched healthy controls. Inclusion criteria were: (i) a documented KS karyotype (47,XXY) for patients and (ii) written informed consent for both groups. KS patients with total testosterone levels lower than 12 mol/l received testosterone replacement treatment according to the current guidelines [15, 16]. Medical students, residents, physicians, nurses, and administrative staff were enrolled as healthy controls. Patients underwent a complete medical and family history evaluation. KS patients and controls were recruited from the 4 Italian centers involved; none of the centers were in high goiter risk areas. Thyroid function and thyroid antibodies (Ab) were assessed. Study participants who had elevated plasma Thyroid Peroxidase (TPO) Ab and Thyroglobulin (Tg) Ab above 350 IU/ml as well as thyroid parenchyma heterogeneity with reduced echogenicity were considered Hashimoto’s thyroiditis (HT) patients. The study was approved by the Ethics Committee of the University of Campania “L. Vanvitelli”–University of Campania L. Vanvitelli” Hospital-AORN Ospedale dei Colli” (no. 1489, 26.10.2015).

Thirty three patients were excluded due to lack of complete data or because they were lost to follow-up.

### Sample collection

Venous blood (2 mL) from study participants was collected from blood banks at KING center hospitals during the same period of KS and control recruitment. Samples, blind of identification, were immediately sent to the laboratory facility in each hospital for analysis of TSH, FT4, FT3, TPOAb, TgAb, and total testosterone. The measurements were done on the same day in primary tubes after blood centrifugation at 3200 rpm for 15 min.

### Biochemical data

As patients attending different centers institutions were recruited, different methods were used to obtain biochemical data. In most cases, serum TSH, FT4, and FT3 concentrations were measured by chemiluminescent immunometric assay (Roche Diagnostics, Mannheim, Germany), as previously described [1]. The manufacturer’s reference limits were: TSH (0.35–5.5 mIU/L), FT4 (10.2–31 pmol/L), and FT3 (3.5–6.5 pmol/L). Detection limit for TSH was 0.005 μIU/mL, and functional sensitivity 0.014 μIU/mL. Thyroid function alteration was classified according to American Thyroid Association guidelines [17]. Clinically overt hyperthyroidism was classified as TSH undetectable to less than 0.1 mIU/liter and FT3 and FT4 above the normal range; subclinical hyperthyroidism as TSH undetectable to less than 0.1 mIU/liter and FT3 and FT4 in the normal range without exogenous T4 intake; clinically overt hypothyroidism as TSH above the upper limit of the normal range (5 mIU/liter in our assay) and FT4 below 10.2 pmol/L; subclinical hypothyroidism as TSH above the upper limit of the normal range and FT4 in the normal range [11, 12]. Total testosterone was measured by immunoassay using a commercially available automated immunoassay system (LIASON Analyzer; DiaSorin, Saluggia, Italy).

### Exclusion criteria

KS patients with mosaic forms of chromosomal aneuploidy or any other structural or numerical karyotype anomaly, with AZF microdeletions were excluded from the study, as previously described [1]. A further exclusion criterion was secondary hypothyroidism defined as TSH undetectable to less than 0.1 mIU/liter and FT4 and FT3 in the lower range without exogenous T4 intake. Enrolled participants with clinically overt thyroid dysfunction or with a personal or family history of thyroid disease were also excluded.

### Thyroid ultrasound and fine needle aspiration

Thyroid US was performed by the same blinded investigator in each center. US and color flow Doppler examinations were mainly performed with a LOGIQ 9 system (GE Healthcare, Chalfont St. Giles, England), a commercially available real-time US system equipped with 5–14 MHz (M12L) and 2.5–7 MHz (7 L) linear array transducers. During the examination, the patient remained in the supine position, and US heads were applied to the right and left side of the thyroid. Four US images were acquired for each participant. The following US characteristics were recorded for each nodule: round shape (ratio of short axis to long axis >0.5), abnormal echogenicity, calcification, cystic aspect, and a peripheral color Doppler pattern.

A conventional 23 G needle was used to collect FNA samples. All sampling procedures were performed by single operator to eliminate operator bias. Sample collection was carried out as follows: (i) neck skin was sterilized with the proper antiseptic; (ii) a clear US view of the most suspicious solid thyroid nodules (based on ACR-TIRADS score) was obtained in the center of the field of view; (iii) each nodule was evaluated in terms of size, vascularity, echogenicity, and calcifications, and data were recorded and archived. FNA was performed when indicated based on nodule characteristics and size following guidelines [17, 18]. Thyroid US was performed by the same blinded investigator in each center in both patients and controls. The same reporting method was used in all centers. Specifically, US risk-stratification system was applied to assess the risk of malignancy and the need for biopsy based on nodule characteristics and size and the data were collected in a standard excel-file used in all the centers.

### Statistical analysis

Data were analyzed using SPSS software (version 17). Mean and standard deviation were calculated for continuous variables. Statistical analyses were performed using Student’s *t*-test. TSH and testosterone levels were compared using Mann–Whitney test, and TPO antibodies using chi-square test.

## Results

A total of 122 KS patients (mean age 37.1, range 18–55) and 85 healthy controls (mean age 39.7, range 18–57) were included in the study. KS patients were confirmed to have FT4 levels in the lower normal range (9.7 ± 3 pg/ml; normal range 7–22 pg/ml) compared to controls (15 ± 8 pg/ml; normal range 7–22 pg/ml). A comparison of the studied variables in KS patients and healthy controls is shown in Table 1. Nodules were observed by US in 31% of KS patients and in 13% of the control group (*p* < 0.01). The maximum diameter of largest nodules size was 22 mm and 19 mm in KS patients and controls, respectively (*p* 0.1), which was not statistically significant (Table 1). The median diameter of the nodules was 7.3 mm and 8.95 mm in KS and controls, respectively.

**Table 1:** Demographic and clinical characteristics of studied population for comparison based on nodular and diffuse thyroid diseases

	KS (*n* 122)	Controls (*n* 85)	*p*
Age (year) (mean ± SE)	37.1 ± 2.3	39.7 ± 1.1	0.1
Prevalence of nodules, (%)	31%	13%	<0.01
Solitary nodule, (%)	53%	58%	0.1
Number of nodules	1.1 ± 0.3	1.3 ± 0.2	0.1
Maximum diameter of largest nodules (mm)	22	19	0.1
Median diameter of nodules (mm) (IQR)^#^	7.3 (5–13)	8.95 (6.7–12.7)	0.1
Moderate and highly suspicious nodules (mean ± SE)	0.26 ± 0.2	0.22 ± 0.2	0.1
Sonographic features of Hashimoto’s thyroiditis	47%	20%	<0.01

The data are presented as mean ± SE

*SE* Standard error of the mean, *IQR* Interquartile range

*P* values calculated using dependent‐samples *t*‐test

^#^Median (IQR)

**Fig. 1:**
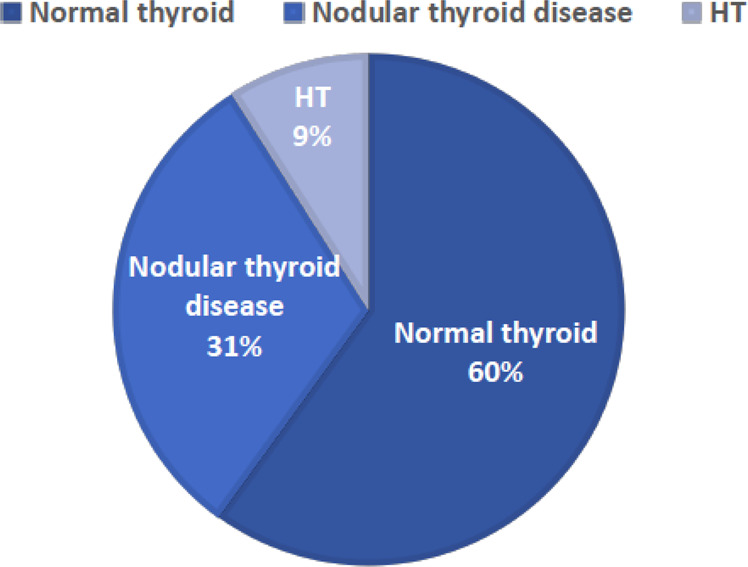
Prevalence of thyroid disease in 122 Klinefelter syndrome patients

Other nodule characteristics, such as number of nodules and prevalence of moderate and highly suspicious nodules in each patient, were similar in both groups. HT was diagnosed in 11 out of 122 KS patients (9%) (Fig. 1) and in 2.5 % in controls. Forty-seven percent of KS patients had US features of HT compared to 20% of controls (*p* < 0.01). A thyroglossal duct cyst was identified in one KS patient. US risk-stratification systems were applied to assess the risk of malignancy and the need for biopsy based on nodule characteristics and size, and FNA was subsequently performed in six out of 37 KS patients and in two controls. All nodules were found to be cytologically benign.

## Discussion

**Fig. 2:**
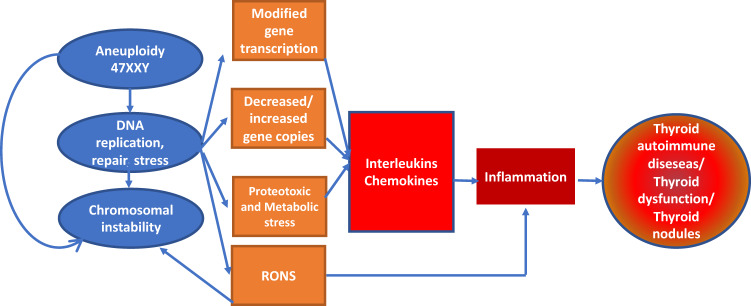
Mechanisms by which aneuploidy may lead to increased inflammation and thyroid dysfunction

The aim of this study was to determine the prevalence of thyroid nodules, as detected by US, in patients with KS compared to healthy controls. Our results show that the prevalence of nodular thyroid disease in KS patients was significantly higher than in male controls (31% vs 13%, respectively). The prevalence of solitary thyroid nodules was similar in the patients and the control group, probably due to the relatively young age of the study population. To our knowledge, this is the first study to address this issue and to present such a finding. A literature search revealed only one report from 1963 describing the incidental finding at postmortem of a nontoxic nodular goiter in a 71-year-old man with KS [12]. In another study assessing thyroid function in patients with aspermiogenesis, one of 12 infertile males treated surgically for nontoxic nodular goiter was found to show an XXY chromosomal constitution [13]. The authors suggest that nontoxic nodular goiter is more frequent in 47XXY than in normal males. More recent data from the literature suggested involvement of hypothalamic–pituitary–thyroid axis dysregulation in adults and children with KS, with a shift toward lower values in the distribution of serum FT4 [1, 11, 14]. To verify this hypothesis, Balercia et al. calculated TSH index, a method for quantitatively estimating the thyrotropic function of the anterior pituitary [19, 20]. Unexpectedly, the authors found no statistical difference in TSH index between KS patients and non-KS patients affected by hypopituitarism. The FT3/FT4 ratio, which indicates peripheral impairment of thyroid hormone conversion and closely correlates with frail status, even in patients presenting free thyroid hormone levels within the reference range was also similar between KS patients and controls [21]. Despite these results and other previously published data, the underlying cause of low FT4 levels in KS remains unclear [1, 11, 14]. Interestingly, there are data showing that individuals with Down syndrome (DS) commonly have TSH levels in the higher normal range and T4 levels in the lower normal range [22–24]. Van Trotesenberg et al. suggested that the mean plasma TSH and T4 levels in DS follow a Gaussian distribution with mean TSH shifted to right and mean T4 shifted to the left, and they considered this phenomenon as a continuum with subclinical hypothyroidism [24]. Surprisingly, this reflects exactly what our and previous studies found in KS. A recent review described the relationship between aneuploidy, inflammation, and diseases, highlighting the need to better understand the emergence of aneuploidy-driven disorders [25]. Chromosome aneuploidy is known to affect cellular function at multiple levels. From higher to lower levels of nuclear organization, gain or loss of chromosome(s) has cytological, molecular, and metabolic effects. Studies comparing several trisomic cell lines showed that despite the variability in chromosome content, aneuploidy triggers uniform transcriptional response in human cells [25]. Imbalance in gene transcription levels will impair protein homeostasis and stoichiometric balance of macromolecular complexes, leading to protein misfolding and aggregation [25]. Interestingly, most data assessing the link between aneuploidy and inflammation were acquired from patients with viable autosomal trisomy or 47, XXY. We previously showed that serum levels of C-C motif chemokine ligand 2 the major chemokine released by monocytes and macrophages and associated with insulin resistance, were higher in testosterone-treated KS patients than in healthy controls [26]. Regarding extra chromosome-driven diseases and thyroid dysfunction, Goswami et al. described two patients with triple X syndrome, both affected by HT, and suggested that this condition may be associated with autoimmune thyroid disorders [27]. We can speculate that the extra chromosome imbalance might be the common factor leading to the higher incidence of autoimmune diseases and inappropriate thyroid function observed in KS, DS, and triple X syndrome (Fig. 2). Further mechanistic and epidemiologic studies are required to obtain a greater insight into the genetic and environmental factors regulating inflammatory responses and hormone pathways. However, our findings could add another piece in the puzzle of aneuploidy-driven disease onset.

One of the strengths of this study is the relatively large sample size of 155 KS patients and 95 age-matched healthy controls. Other important strengths are that the study used blinded investigators to perform thyroid ultrasounds and fine needle aspirations, which reduces the potential for bias in the interpretation of the results and that established guidelines were used to classify thyroid function alterations, Hashimoto’s thyroiditis and to characterize thyroid nodules. There are, however, some limitations to be considered. First, the study was a case-control study, which can only establish an association and not causation between KS and thyroid disorders. The study used different methods to obtain biochemical data, which may introduce some variability in the results

In conclusion, this study showed for the first time that patients with KS have higher prevalence of nodular thyroid disease in comparison with matched controls. The increased risk of nodular thyroid disease in KS is likely linked to low levels of FT4, inappropriate TSH secretion, and/or genetic instability. However, further studies are needed to confirm these hypotheses.
